# Why does the Conductivity of a Nickel Catalyst Increase during Sulfidation? An Exemplary Study Using an *In Operando* Sensor Device

**DOI:** 10.3390/s151027021

**Published:** 2015-10-23

**Authors:** Peter Fremerey, Andreas Jess, Ralf Moos

**Affiliations:** 1 Department of Functional Materials, Zentrum für Energietechnik (ZET), University of Bayreuth, 95440 Bayreuth, Germany; E-Mail: functional.materials@uni-bayreuth.de; 2 Department of Chemical Engineering, Zentrum für Energietechnik (ZET), University of Bayreuth, 95440 Bayreuth, Germany; E-Mail: Andreas.Jess@uni-bayreuth.de

**Keywords:** fixed bed catalyst, sulfur poisoning, nickel to nickel sulfide transformation, percolation, in operando

## Abstract

In order to study the sulfidation of a catalyst fixed bed, an *in operando* single pellet sensor was designed. A catalyst pellet from the fixed bed was electrically contacted and its electrical response was correlated with the catalyst behavior. For the sulfidation tests, a nickel catalyst was used and was sulfidized with H_2_S. This catalyst had a very low conductivity in the reduced state. During sulfidation, the conductivity of the catalyst increased by decades. A reaction from nickel to nickel sulfide occurred. This conductivity increase by decades during sulfidation had not been expected since both nickel and nickel sulfides behave metallic. Only by assuming a percolation phenomenon that originates from a volume increase of the nickel contacts when reacting to nickel sulfides, this effect can be explained. This assumption was supported by sulfidation tests with differently nickel loaded catalysts and it was quantitatively estimated by a general effective media theory. The single pellet sensor device for *in operando* investigation of sulfidation can be considered as a valuable tool to get further insights into catalysts under reaction conditions.

## 1. Introduction

Sulfur is one of the best known poisons for many industrial catalysts, which deactivates catalysts already in small concentrations [1]. Up to now, no direct method has been known to detect sulfur poisoning of a fixed bed *in operando* during the process. Classical *ex situ* methods, however, to monitor the sulfur content or models to estimate it, do not allow an immediate response. 

In [2], a resistive (or impedancemetric) sensor was designed to detect coking and decoking *in operando* directly in the refinery process. A single pellet of the fixed catalyst was considered as an exemplary representative. It was contacted with electrodes, and its resistance or impedance was measured. A strong correlation between coking degree and single pellet (sensor) impedance proved the suitability of this concept. 

In a previous study, we showed initial data how the same principle can be also applied for sulfidation detection of commercial silica pellets loaded with highly dispersed nickel (commercial refinery catalysts for gasoline reforming) [3]. Since H_2_S chemisorbs on the catalyst surface and forms nickel sulfides, deactivation of the active centers follows [4,5,6,7]. In this study, an interesting effect was observed: Despite a high nickel loading of circa 40 wt%, the single pellet remained electrically insulating. However, with proceeding sulfidation, the sensor resistance decreased by decades. This is the more astonishing, as the formed sulfides are not that good conducting than metallic nickel is. 

The nickel catalysts used in the present study are cylindrically shaped porous fixed bed particles (“pellets”) with a diameter of 6 mm and a length of also 6 mm. Compared to coke, sulfur does not get deposited on the catalyst surface but reacts with the active centers of the catalyst. Therefore, the catalyst itself is at first examined in more detail (Section 2). After that, the sensing mechanism is a subject of the study. In short, this study investigates why the conductivity of a nickel catalyst increases during sulfidation by applying the investigated *in operando* sensor device.

## 2. Experimental Setup

**Figure 1:**
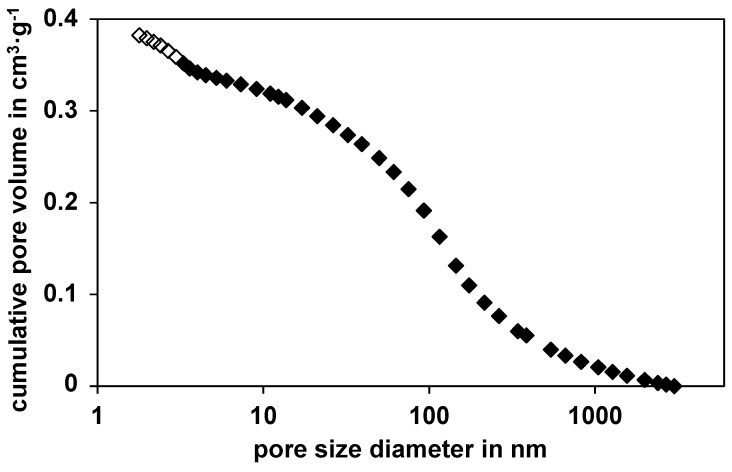
Cumulative pore volume of the nickel catalyst against the pore diameter based on the BET analysis (white rhombi) and the mercury-porosity measurement (black rhombi) Data from [8].

The used commercial nickel catalyst (NiSAT 200) was characterized by SEM and EDX (energy dispersive X-ray spectroscopy). In addition to silicon and nickel, also calcium and aluminum are found by EDX on the catalyst surface. This agrees with the manufacturer’s data sheet (Clariant, former SüdChemie), which specifies the composition to be between 25 wt% and 50 wt% nickel monoxide, below 25 wt% calcium aluminate and more than 20 wt% silica. Also, the pellets should contain less than 5 wt% graphite. Other parameters such as the BET surface area (100 m^2^/g), the catalyst density (1.515 g/cm^3^), the average pore diameter (7 nm) and the porosity (0.54) were measured in our labs (N_2_-physisorption was used to determine the BET (Brunauer, Emmett and Teller) surface and the average pore diameter is calculated through the adsorption curve; used device: Micromeritics series Gemini 2375 (Micromeritics, Norcross, GA, USA)). To obtain catalyst density, the volume of 20 particles (diameter, height), and their weight were measured and averaged. The porosity was calculated through the dry and wet mass of these single catalyst particles. The pore size distribution of the catalyst is shown in Figure 1. It should be noted at this point, that even after sulfidation, the BET surface and the pore size distribution, remained constant.

**Figure 2:**
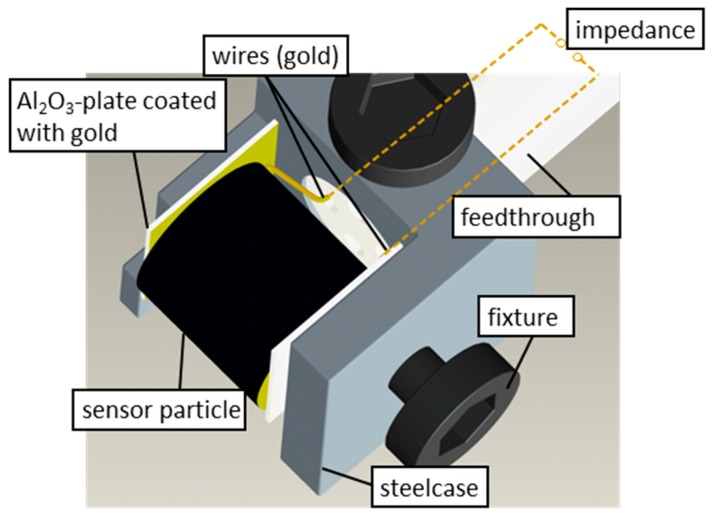
Illustration of the sensor device. Modified after [3].

The catalyst pellets of the sensors were contacted electrically at the flat end planes and were fixed in a steel casing. The contacted gold wires were fed out of a thermally isolated quartz glass reactor via a ceramic tube. Inside of this reactor, the single pellet sensor was fixed in a glass holder. Figure 2 illustrates the sensor setup. A more detailed description of the single catalyst sensor device and the lab test bench is given in [3]. The gas flow for the sulfidation experiments was 50 L/h (NTP) with an H_2_S concentration of up to 1000 ppm in N_2_. 

The single pellet sensor was connected with an impedance analyzer (HP 4284A Precision LCR meter, Agilent Technologies, Santa Clara, CA, USA). For the below-given mathematical analysis of the sulfidation experiments, the effective conductivity, *σ*, of the sensors was calculated from the complex impedance, measured at 1 kHz, under the assumption of an R||C equivalent circuit. The effective conductivity, *σ*, was derived from the resistance *R* with the geometry of the catalyst particle. For further details see [3]. 

Prior to the sulfidation experiments, the single catalyst sensor was heated to 400 °C under N_2_. After this temperature had been established, the catalyst was reduced under 10% hydrogen in N_2_ for 1 h, since it cannot be excluded that the nickel existed (at least partly) in its oxide form. In the presence of nickel oxide, the active pure nickel may have reacted (partly) with sulfur to nickel sulfate. This sulfate formation would have complicated the chemical reactions, and secondary effects might have affected the sensor response.

At the last step prior to sulfidation, the reactor was cooled down to the desired temperature, again under N_2_. As soon as the conductivity had reached a stable value, the sulfidation began. This preconditioning procedure was conducted with each single pellet sensor device to establish a defined pristine state.

## 3. Basic Results

**Figure 3:**
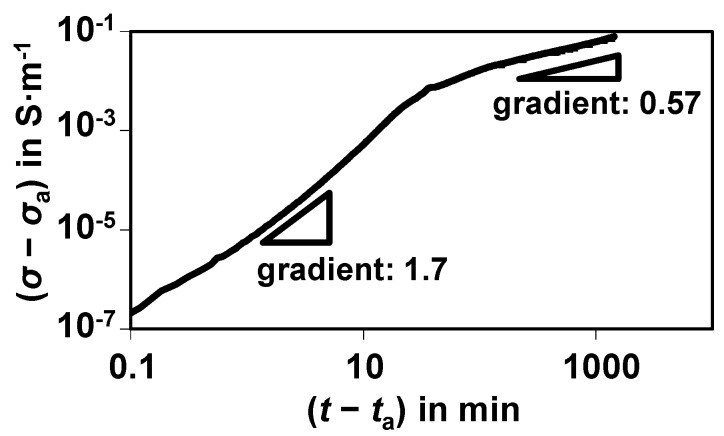
Electrical response (conductivity change *σ* − *σ*_a_) during sulfidation of a single nickel catalyst pellet at 300 °C with 500 ppm H_2_S in N_2_.

A typical result for a sulfidation experiment at 300 °C with 500 ppm H_2_S in N_2_ in the feed is shown in Figure 3. It is already corrected by the dead time of the sensor test bench, *t*_a_. To show the conductivity increase *σ* − *σ*_a_, the starting value without sulfur, σ_a_, was already subtracted. The conductivity increase is plotted in a double-logarithmic representation. 

Besides the huge increase in the conductivity by decades, which will be discussed below, two slopes in the curve become obvious. They may stem from different physical processes. The slope in the double-logarithmic representation is approximate 1.7 when the sulfidation starts and decreases to only 0.57 after about 1 h. According to [9], a value in the order of the second slope can be attributed to a diffusion process. 

At the beginning of the sulfidation, the catalyst has a low conductivity σ_a_ in the reduced state. Since the highly dispersed small nickel particles do not have electrical contact to each other, this low value may mainly originate from the thermally activated conductivity of the insulating oxide ceramic carrier pellet. When the catalyst gets sulfidized, the nickel particles react with the hydrogen sulfide to form nickel sulfides in several stoichiometries with the end member NiS (XRD (X-Ray diffraction) and EDX analysis confirm that [3]). All of these nickel sulfides show the character of a metallic conductor [10,11,12] but with a markedly lower conductivity than pure nickel. 

**Figure 4:**
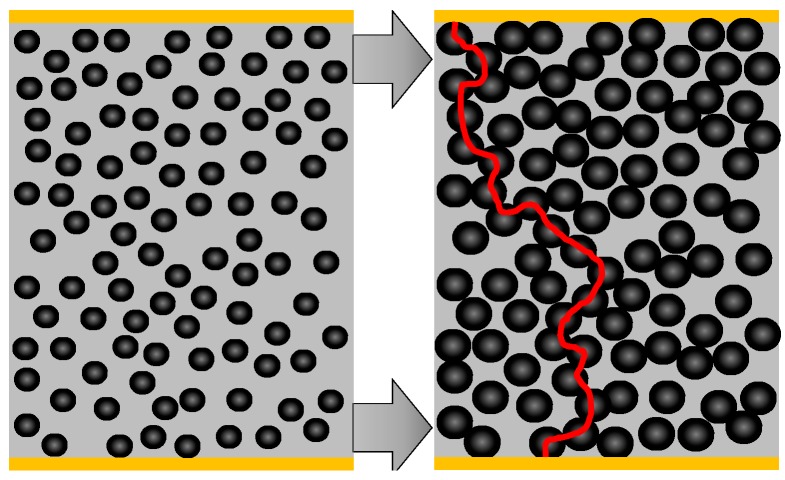
Scheme to illustrate how the volume increase during sulfidation of the dispersed nickel catalyst influences the electrical conductivity.

With the reaction from nickel to nickel sulfide, the highly dispersed nickel grains increase their volume (which is exemplary depicted in Figure 4). This is a first qualitative explanation of the strong conductivity increase.

On the left side of Figure 4, a catalyst particle in the reduced state is depicted. The nickel grains are dispersed and they do not have electrical contact amongst each other, at least there is no continuous conductive path from one electrode to the other. Since no highly conductive paths either from Ni or from NiS exist between the electrodes, only the catalyst support, which is not ideally insulating due to its oxide character, may contribute to an electrical current. If the particles get sulfidized and nickel sulfides form, their volume increases by a factor of about 2.5 [13]. This is illustrated on the right side of Figure 4. The newly formed conductive path is marked by a red line. Please note that in reality more than one conductive path will be formed. Such a phenomenon can be explained by the percolation theory. Due to the volume increase of the conductive material (as it is the case when nickel sulfides are formed), the so-called percolation threshold is exceeded and the conductivity of the catalyst increases strongly. According to [14,15], a slope of about 1.7 is in the range of a typical percolation process in thin films. This is a strong hint that the observed conductivity increase can be attributed to a percolation process. Since in the catalyst pellets, nickel is present in very thin films, the assumption of a 2D percolation seems justified. To obtain a more quantitative model, the increasing conductivity shall be described by a General Effective Media Theory (GEMT), which is an extension of the percolation theory [16]. It reproduces even better the entire range including the smaller conductivity increase when many percolation paths exist [17]. For the GEMT, the conductivity must be plotted over the volume content of the conductive phase, *Φ*. In our experiment, the samples are sulfidized at 300 °C with a H_2_S concentration of 500 ppm. According to [1], a shrinking core model explains the timely behavior of the sulfidation. In [3], this assumption has been quantitatively validated and the shrinking core model was plotted against the time. Additionally, the conductivity measurement in this paper is plotted against the time. Both measurements are calculated for the same time interval and the conductivity was plotted against the NiS volume content, *Φ*, derived from the shrinking core model. With these parameters, the GEMT model according to Equation (1) can be applied [17]. Equation (1) has to be solved numerically.

(1) $$0\;=\;\frac{\left(1\;-\;\Phi \right)\cdot \left(\sigma_{L}^{\frac{1}{\omega }}\;-\;\sigma_{\text{total}}^{\frac{1}{\omega }}\right)}{\sigma_{L}^{\frac{1}{\omega }}\;+\;\left(\frac{1\;-\;\Phi_{c}}{\Phi_{c}}\right)\cdot \sigma_{\text{total}}^{\frac{1}{\omega }}}+\frac{\Phi \cdot \left(\sigma_{H}^{\frac{1}{\omega }}\;-\;\sigma_{\text{total}}^{\frac{1}{\omega }}\right)}{\sigma_{H}^{\frac{1}{\omega }}\;+\;\left(\frac{1\;-\;\Phi_{c}}{\Phi_{c}}\right)\cdot \sigma_{\text{total}}^{\frac{1}{\omega }}}$$

**Figure 5:**
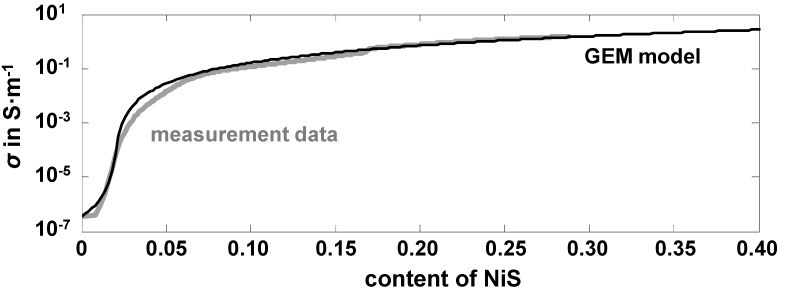
Measured effective conductivity and the calculated values from the GEMT model, plotted over the content of the converted nickel to nickel sulfide; Experimental data: *T* = 300 °C, 500 ppm H_2_S in N_2_, *V* = 50 L·h^−1^ (NTP), *p* = 1 bar.

In Equation (1), *Φ* is the volume content of the highly conductive phase, NiS. For instance, *Φ* = 1 means that all Ni is converted into nickel sulfides. Other parameters are the critical percolation volume part of the highly conductive phase, *Φ*_c_, and the model parameter ω, which is also called the critical exponent. *σ*_H_ (index “H” for “high”) is the effective conductivity of the catalyst when all Ni is converted into nickel sulfides. It is determined by the interpolation of the conductivity curves up to a full conversion. *σ*_L_ (for “low”) denotes the effective conductivity in the reduced state, which is in our case the low conductivity of the oxide catalyst pellets. *σ*_total_ represents the conductivity as it is calculated by the GEMT during the sulfidation process. Now *Φ*_c_ and *ω* can be fitted so that the model correlates with the measured curve. A good agreement between the measured conductivities and Equation (1) can be obtained for *Φ*_c_ = 0.02 and *ω* = 1.8. According to these model parameters, only a minimal amount of sulfur is needed (namely 2%) until the percolation threshold is reached. We assume that this very small critical concentration is due to the interaction between the thin film percolation and the sulfur loading after the shrinking core model. The values for the parameter ω coincides with other modeled curves for three-dimensional figures [16]. Figure 5 compares measurement and calculation.

The good agreement between both curves allows (at least formally) to apply the GEMT and to attribute the strong conductivity increase to percolation paths that are formed during sulfidation. This agrees with older data of [18] for commercial catalysts consisting of nickel on an alumina-based carrier material.

Percolation phenomena can be found in many other sensor and catalyst systems. Some literature studies report on the percolation of metals in insulators [19,20,21,22,23]. In [24], similar results for a gas dosimeter-type accumulative hydrogen sulfide gas sensor are obtained. Copper oxide nanofibers are converted into copper sulfide. Depending on the amount of converted copper, sudden contact between the nanofibers is formed. As soon as the percolation threshold is reached, an abrupt increase of the conductivity is found, similar as in [17]. Furthermore, [25,26] found such an accumulative percolation behavior for conductometric NO_x_ sensors when rare earth metals are transformed from carbonates into nitrates.

Section 4 below intends to estimate numerically the volume increase and its effect on the conductivity. In particular, it shall be shown that the amount of formed nickel sulfide and the corresponding volume increase suffices to exceed the percolation threshold. The reader may skip Section 4 and go directly to the experimental Section 5, where it will be shown how and when the percolation paths are formed for different nickel contents.

## 4. Estimation of the Nickel Grains on the Catalyst Particle

The volume increase of nickel when getting transformed into nickel sulfide and the question of whether the percolation threshold can be reached just by this volume increase is discussed in this section.

**Figure 6:**
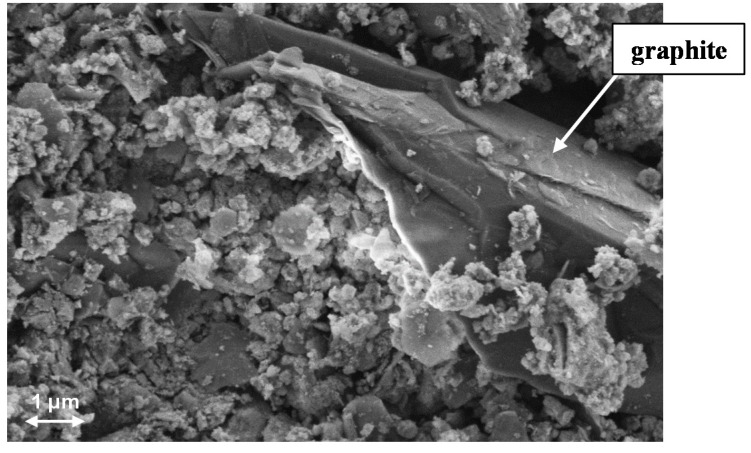
SEM image from the catalyst surface.

The BET surface area was determined to approximately 100 m^2^·g^−1^. Since the catalyst contains 5 wt% graphite, graphite grains can be detected by SEM and EDX on the catalyst surface (Figure 6). 

Therefore, in addition to the nickel grains, already 10% of the total surface area is filled with a conductive material even before the sulfidation starts. This percentage was subtracted from the original BET surface area. The nickel surface area is determined by adsorption and desorption of hydrogen during TPD experiments. If one assumes that each hydrogen atom adsorbs on one nickel atom, the measured 272 µmol H_2_ per gram catalyst yield to the nickel surface per gram of about 21.3 m^2^·g^−1^ (the Avogadro constant of 6.022 × 10^23^ parts per mol and the cross-sectional area of nickel atoms of 0.065 nm^2^ was used). With ICP-MS (inductively coupled plasma mass spectrometry) measurement, the nickel content was determined to be circa 40 wt%. The volume of the nickel per gram catalyst can be determined by this nickel content and the density of nickel (8.91 g·cm^−3^). If it is further assumed that nickel is available in the form of spherical grains on the catalyst surface, the calculation of the diameter leads to the relationship between nickel surface area (πd^2^) and the nickel volume ($\frac{1}{6}{\pi d}^{3}$):

(2) $$\frac{A}{V}\;=\;\frac{6}{d}$$

This gives an average diameter for the nickel grains of about 12.7 nm. All obtained data are summarized in Table 1.

**Table 1:** Measured parameters and the resulting average diameter of the nickel spherical grains on the catalyst surface.

Aim	Realization	Result
determination of surface from a catalyst pellet	BET measurement	100 m^2^·g_Kat_^−1^
determination of the nickel surface	TPD experiment: H_2_ adsorbs at nickel	21.3 m^2^·g_Kat_^−1^
determination of the nickel content	ICP measurements at single particles	40 wt%
calculate the average diameter of a nickel grain	calculate the available nickel volume	4.5 × 10^−8^ m^3^·g_Kat_^−1^
	calculate the diameter with the metal surface and the volume	12.7 nm

**Figure 7:**
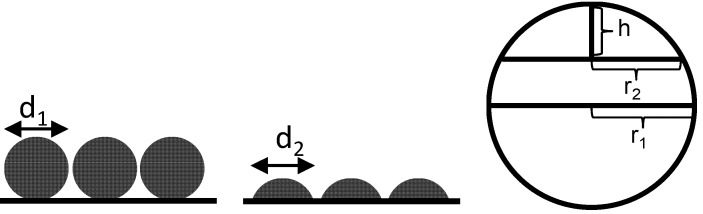
Schematically correlation of spherical grains and spherical calottes.

However, it is expected that no complete spherical grains are on the surface but rather spherical calottes instead. The total number for such spherical calotte depends on the selected calotte height. For a decreasing calotte height, the number of spherical calottes increases, but the surface of each calotte decreases. In Figure 7, this relationship is demonstrated.

For the calculation of the calotte number, the circular base area of the spherical calottes is not counted for the surface. This area is not accessible and is also not measured in the TPD experiments. Therefore, for the surface of the spherical calottes, *A*, Equation (3) can be used [27]:

(3) $$A_{\text{spherical}\;\text{calotte}}\;=\;2\pi \cdot r_{1}\cdot h$$

For a spherical calotte of the mean sphere with a height of *h* = 1 nm, this calculation gives 5.34 × 10^17^ spherical nickel calottes. The assumption of a height of 1 nm seems rather arbitrary but with a resulting diameter of 12.7 nm, it seems to be justified. Nevertheless, one should consider this entire section only as a rough estimation that percolation by volume increase may explain the observed conductivity increase by decades. 

**Figure 8:**
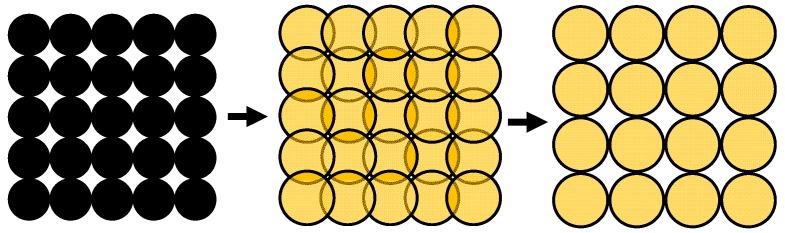
The theoretical full load with nickel grains on the left side. During sulfidation, the circular areas increase (in the middle) and a smaller number of spherical grains are needed for the theoretical full load (on the right side).

In the next step, the area which is occupied through this spherical calottes can be specified, when considering the BET surface as a rectangular planar surface. The considered area is 90 m^2^·g^−1^ and the length and width are approximately 9.49 m per 1 g catalyst. The following calculation considers the circular geometry of the spherical calottes to estimate a loaded percentage. This means that the number of spherical calottes will be determined that would be necessary to cover the catalyst surface fully. Finally, this maximum number is compared with the calculated number. The quotient between these two numbers gives an estimation for the percentage load of the surface. When sulfidation proceeds, the spherical calottes increase, but their number remains the same. In contrast, the number of the theoretically calculated full load decreases due to the volume increase. If the existing number is now compared with this theoretical full load, the percentage of the occupied conductive part increases. Figure 8 illustrates this process.

For the pure nickel, a theoretical full load is reached at 1.95 × 10^18^ spherical calottes, representing a loading of 27.4% for the calculated 5.34 × 10^17^ spherical nickel calottes. For the sulfidized spherical calottes (with a theoretical full load of 1.02 × 10^18^), the loading corresponds to approximately 52.4%. 

These calculated loading levels can now be compared with typical percolation thresholds from literature. The percolation threshold of a simple cubic lattice is *ca.* 31% for a site percolation and *ca.* 25% for a bond percolation [28]. Both values are exceeded in the sulfidized case. Even the pure nickel catalyst is close to the percolation threshold. The assumption of the percolation threshold of a cubic lattice is legitimated, since the value of the measured BET surface is from the three-dimensional structure of the catalyst particle. 

This calculation can only be a rough estimation, for an exact calculation, many more points would have to be considered. A certain part of the surface was ignored when using round solids and not square solids. A circle captures only 78.5% of the rectangle area (edge length is equal to diameter). Any other geometries such as rod-shaped nickel grains are ignored in this calculation. From EDX analysis, we know that nickel is not homogeneously distributed over the catalyst surface. Other effects, like the tunnel effect [29] are also important, since then nickel grains or nickel sulfide grains may not need full contact. All of these effects are not considered in the calculation, but they would lead to an earlier conductivity increase compared to the pure contact calculation.

All in all, these rough estimations support the percolation approach, *i.e.*, the assumption that conductive paths are formed on the catalyst during sulfidation can also be quantitatively estimated.

## 5. Further Measurements to Support the Estimation

Since all previous observations and calculations in Section 3 as well as the quantitative estimations in Section 4 support the assumption that conductive percolation paths form as a result of the volume increase during sulfidation, we conducted further experiments that strongly corroborate our assumption. 

For that purpose, a catalyst with different nickel contents was prepared. It was loaded with different nickel contents to find out which amount of nickel is required to exceed the percolation threshold when sulfidized with hydrogen sulfide. An alumina catalyst (length and diameter of the catalyst pellets are 5 mm) with a BET surface of about 230 m^2^·g^−1^ was used as a carrier support. This carrier support was soaked with a nickel nitrate solution (20 wt% of nickel in water; prepared from nickel nitrate hexahydrate dissolved in water) under vacuum (50 mbar) to be sure that all pores of the catalyst pellets were filled with the solution. Finally, the pellets were calcined in synthetic air. The samples were heated with a very slow ramp of 2 °C/min to the calcination temperature of 350 °C. Such a slow heating process supports the formation of small nickel clusters [30]. During the two hour calcination, nickel was deposited as nickel oxide on the carrier support. After that, some prepared particles were retained for sulfidation tests and the same procedure was repeated with the remaining particles for several times until the nickel content of the self-prepared nickel catalyst reached the value of the industrial NISAT 200 catalyst. By this procedure, a series of catalysts pellets with slowly increasing nickel content was obtained. All prepared catalysts were electrically contacted as shown in Figure 2 and sulfidized. Their conductivity was recorded during sulfidation as described in the experimental section. 

In Table 2, the parameters for the tests with the self-prepared nickel catalyst are listed. The sample with number 0 represents the pure alumina supported catalyst. The nickel content increased as a result of the described procedure.

**Table 2:** Data from the self-prepared catalyst with different nickel contents on an Al_2_O_3_ carrier support.

Sample	Nickel Content (wt%)	Bet Surface (m^2^·g^−1^)	Sample Mass (g)	Calculated Area (m^2^)
0	0	230	0.11	25
1	7.4	229	0.12	27
2	14.9	205	0.13	27
3	19.7	189	0.13	25
4	24.6	180	0.15	27
5	30.9	153	0.15	23
6	32.2	147	0.16	24
7	36.4	152	0.17	26
8	37.5	139	0.19	26
9	40.1	127	0.19	24
10	41.3	135	0.20	27

The nickel content was determined on calcined particles by ICP-MS measurements under the assumption that nickel exists as nickel oxide. For the first two nickel loading steps, the nickel content increased constantly. After that and especially from a nickel content above about 30 wt%, the amount of nickel that could be deposited on the catalyst decreased with each loading step. This effect, however, shall not be considered further; for the sulfidation experiments, it is important that catalysts with different nickel loadings could have been prepared. 

The BET surface decreased from 230 m^2^·g^−1^ for the pure carrier support to about 130 m^2^·g^−1^ for the highly nickel loaded samples. This decrease, however, only seems that way on the surface. The area of an individual pellet remained nearly constant, but due to the mass increase (higher nickel content; as described in Table 2), a lower BET surface results. Therefore, a blocking of the pores did not occur. 

**Figure 9:**
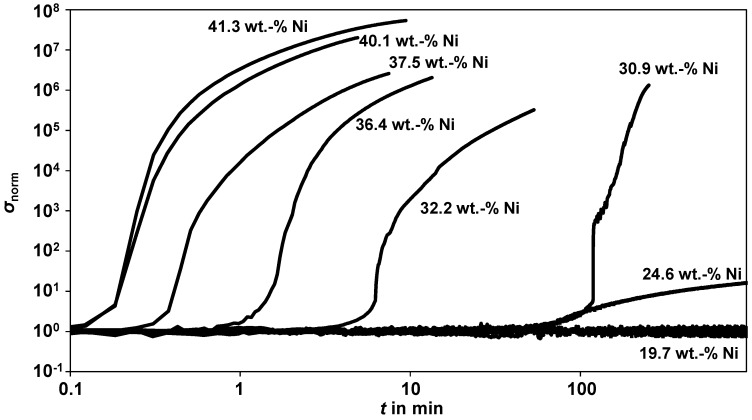
Normalized conductivity during sulfidation of self-prepared single catalyst pellets with different nickel contents. The curves from 0 wt% Ni to 32.2 wt% Ni were smoothed over 10 measurement points; Experimental data: *T* = 400 °C, 1000 ppm H_2_S in N_2_, *V* = 50 L·h^−1^ (NTP), *p* = 1 bar.

These samples were sulfidized and measured under the same conditions as described above. To obtain faster data, 1000 ppm H_2_S in N_2_ were used (instead of 500 ppm) and the temperature was increased to 400 °C. The conductivity data are shown in Figure 9. For better comparison, the conductivity, σ, was normalized to the value in the non-sulfidized state, *σ*_a_, taken right before the sulfidation started, *σ*_norm_ = *σ*/*σ*_a_. 

For the pure carrier and the Samples 1 to 3 (up to 19.7 wt% nickel), no conductivity increase occurred until the end of the sulfidation at 15 h. Since these curves were almost identical, only the curve for the nickel content of 19.7 wt% (Sample 3) is plotted. For Sample 4, which has a nickel content of approximately 24.6 wt%, a minimal increase of the conductivity can be seen after 80 min. Not before a nickel content of 30.9 wt% (Sample 5) was reached did the strong, step-like conductivity increase occur. The higher the nickel content, the earlier this sudden conductivity increase began. For the catalyst with about 40 wt% nickel, the percolation threshold was reached already after some tens of seconds. 

Figure 9 clearly shows that a certain amount of nickel is required before the conductivity increase sets in. Obviously, in the 24.6 wt% nickel sample, the percolation threshold is not reached and no conductive paths are formed. We assume that the single nickel sulfide clusters are still (almost) insulated from each other and the volume increase contributes only a small extent to the sensor signal. When the nickel content reaches 30 wt%, the percolation threshold can be exceeded during sulfidation. With a further increasing nickel content, the percolation threshold is reached even faster, because only a smaller amount of sulfur is required until the first percolating paths are formed. Even though the self-prepared single catalyst pellets differ in some points from the commercial nickel catalyst used in this study (e.g., carrier material, BET surface, and different nickel dispersion), the percolation threshold is reached at the same nickel content range during sulfidation. This supports the previous conclusions.

The percolation hypothesis was checked through a final experiment with the industrial nickel catalyst (NiSAT 200). It should prove that its nickel content is close the percolation threshold. For that purpose, the nickel content of the NiSAT catalyst was stepwise increased, by the identical procedure as described above for the alumina catalyst. The nickel content was again determined by ICP-MS. Again, the catalyst pellets were reduced before sulfidation started. The respective conductivity values *σ*_a_ are summarized in Table 3. 

**Table 3:** Results of the commercial nickel catalyst with additional nickel content; measurement at *T* = 450 °C, *V* = 50 L·h^−1^ (NTP) N_2_, *p* = 1 bar.

Sample	Nickel Content (wt%)	Conductivity, *σ*_A_, Reduced at 450 °C; (S·m^−1^)
(0) pure catalyst	40.3	10^−5^–10^−7^
(1) catalyst + Ni	45.1	2.7 × 10^−6^
(2) catalyst + Ni	49.1	1.7 × 10^−5^
(3) catalyst + Ni	51.3	1.3 × 10^−4^
(4) catalyst + Ni	55.7	1.84

Generally, it can be obtained from Table 3 that the conductivity in the reduced but unsulfidized state increased with higher nickel content. Between 50 wt% and 55 wt%, however, a strong increase by about five decades occurs. Here, enough nickel is loaded on the catalyst to enable percolation paths already without any sulfidation. 

All of these results obtained by the *in operando* sensor device have proven that the increase of the conductivity of the nickel catalyst is due to the formation of percolating paths that occurs during the sulfidation reaction of nickel to nickel sulfide. Furthermore, the commercial nickel catalyst has a nickel content, which is very close to this percolation threshold. Since it is clear that the heterogeneously catalyzed reactions always occur on the surface of the catalyst, especially on the surface of the noble metal components, a high dispersion is essential for good catalytic properties. In order to avoid “unused” atoms inside the particles [1], usually very small particles of the active component (often a metal or metal oxide) are deposited on a carrier having a high specific surface and the metal content of an industrial catalyst is therefore often close to the percolation threshold to ensure a high conversation rate. This has now been verified by the electrical sensor experiments.

## 6. Conclusions and Outlook

Altogether, all conducted experiments as well as the calculations and estimations indicate that the electrical conductivity increase during sulfidation of these catalysts can be explained by the formation of conductive percolation paths as a result of the volume increase of nickel when getting transformed to nickel sulfide. To reach the percolation threshold, only a small amount of sulfur is needed since the catalysts show a high degree of dispersion and the nickel loading is close to the amount of that which is necessary to reach the percolation threshold. At the beginning of the sulfidation, the formation of the conducting paths can be described by the General Effective Media Theory. Later, a diffusion mechanism that follows the shrinking core model prevails. The single pellet sensor for *in operando* investigation of sulfidation can be considered as a valuable tool to measure the sulfidation of the catalyst under reaction conditions.
